# Competing Endogenous RNA in Colorectal Cancer: An Analysis for Colon, Rectum, and Rectosigmoid Junction

**DOI:** 10.3389/fonc.2021.681579

**Published:** 2021-06-10

**Authors:** Lucas Maciel Vieira, Natasha Andressa Nogueira Jorge, João Batista de Sousa, João Carlos Setubal, Peter F. Stadler, Maria Emília Machado Telles Walter

**Affiliations:** ^1^ Departamento de Ciência da Computação, Instituto de Ciência Exatas, University of Brasília, Brasília, Brazil; ^2^ Bioinformatics Group, Department of Computer Science, and Interdisciplinary Center for Bioinformatics, Leipzig, Germany; ^3^ Division of Coloproctology, Department of Surgery, University of Brasília School of Medicine, Brasília, Brazil; ^4^ Department of Biochemistry, Institute of Chemistry, University of São Paulo, São Paulo, Brazil; ^5^ Max Planck Institute for Mathematics in the Science, Leipzig, Germany; ^6^ Institute for Theoretical Chemistry, University of Vienna, Wien, Austria; ^7^ Facultad de Ciencias, Universidad National de Colombia, Sede Bogotá, Colombia; ^8^ Santa Fe Institute, Santa Fe, CA, United States

**Keywords:** colorectal cancer, competing endogenous RNA, TCGA, long non-coding RNA, miRNA, mRNA

## Abstract

**Background:**

Colorectal cancer (CRC) is a heterogeneous cancer. Its treatment depends on its anatomical site and distinguishes between colon, rectum, and rectosigmoid junction cancer. This study aimed to identify diagnostic and prognostic biomarkers using networks of CRC-associated transcripts that can be built based on competing endogenous RNAs (ceRNA).

**Methods:**

RNA expression and clinical information data of patients with colon, rectum, and rectosigmoid junction cancer were obtained from The Cancer Genome Atlas (TCGA). The RNA expression profiles were assessed through bioinformatics analysis, and a ceRNA was constructed for each CRC site. A functional enrichment analysis was performed to assess the functional roles of the ceRNA networks in the prognosis of colon, rectum, and rectosigmoid junction cancer. Finally, to verify the ceRNA impact on prognosis, an overall survival analysis was performed.

**Results:**

The study identified various CRC site-specific prognosis biomarkers: hsa-miR-1271-5p, *NRG1*, hsa-miR-130a-3p, *SNHG16*, and hsa-miR-495-3p in the colon; *E2F8* in the rectum and *DMD* and hsa-miR-130b-3p in the rectosigmoid junction. We also identified different biological pathways that highlight differences in CRC behavior at different anatomical sites, thus reinforcing the importance of correctly identifying the tumor site.

**Conclusions:**

Several potential prognostic markers for colon, rectum, and rectosigmoid junction cancer were found. CeRNA networks could provide better understanding of the differences between, and common factors in, prognosis of colon, rectum, and rectosigmoid junction cancer.

## Introduction

Colorectal cancer (CRC) is one of the most common and lethal cancers in the world (1). The most common histopathological type of CRC is adenocarcinoma (2). CRC can be classified according to its three major affected sites: colon, rectum, and rectosigmoid junction. Together these make up the large bowel. The colon corresponds to the largest portion, the rectum is located at the end, and the rectosigmoid junction is the transition between colon sigmoid and rectum. A tumor site is classified as belonging to the rectosigmoid junction when differentiation between rectum and sigmoid is not possible (3). CRC tumor site identification is important, due to different treatment strategies: for the colon, radical resection, depending on the stage, combined with chemotherapy is used; for rectum, only radical surgery or neoadjuvant chemorradiation followed or not by radical resection (4); while for the rectosigmoid junction, the best treatment still remains unknown (5). Because erroneous diagnosis of the CRC site can lead to overtreatment with chemotherapy, the identification of the CRC tumor site is a process that should be carefully analyzed, especially in the rectosigmoid junction (4). Better understanding of the biological characteristics of CRC at each site may provide insight into its development and progression.

Many studies have highlighted the importance of long non-coding RNAs (lncRNAs) in understanding the biological mechanisms of CRC and other types of cancer. LncRNAs are molecules greater than 200 nucleotides that do not encode for proteins and act mainly as transcriptional regulators by interacting with other molecules, such as miRNAs and mRNAs (6). These interaction mechanisms between lncRNAs and other molecules are explained by the competing endogenous RNA (ceRNA) hypothesis, which describes the interactions and their influence on altered protein expression levels (7). CeRNA network analyses have reported differentially expressed lncRNAs involved in breast, gastric, and many other types of cancer (8–10). Specifically for CRC, many studies also analyzed the ceRNAs networks and indicated potential diagnosis and prognosis biomarkers for colon, rectal, and colorectal cancer in general (1, 11–16). Most of these studies investigated the ceRNAs of CRC without differentiating the anatomical sites; however, different CRC sites present unique characteristics and treatment responses. Therefore, the identification of exclusive biomarkers for colon, rectum, or rectosigmoid junction could aid in understanding differences in the disease prognosis and progress. To the best of our knowledge, our study is the first to establish specific ceRNA networks for (i) colon; (ii) rectum; and (iii) rectosigmoid junction, and to associate them with specific biological mechanisms in order to clarify the differences and common factors between these sites.

In this study, we analyzed the functional and prognostic roles of lncRNAs, miRNAs, and mRNAs in colon, rectum, and rectosigmoid junction cancer, based on specific ceRNA networks constructed by using data from The Cancer Genome Atlas (TCGA) rectal adenocarcinoma (READ) and colon adenocarcinoma (COAD) projects.

## Materials And Methods

### Data

The RNA expression value raw count and clinical information data of patients with colon, rectum, and rectosigmoid junction CRC sites were downloaded from TCGA (17). The selection criteria was: (1) open access to information; (2) sample types from primary tumor or solid normal tissue; (3) patients with adenocarcinomas.

### Analysis of Differentially Expressed RNAs

We used the GDCRNATools v1.6 (18) R package and implemented the limma (19) method to obtain differentially expressed (DE) lncRNAs, miRNAs, and protein coding genes (*PCGs*) for cancer and normal tissue. The expression profiles were normalized by the voom method implemented in the GDCRNATools. The RNAs presenting FDR ≤ 0.05 and |logF C| ≥ 2 were considered statistically significant.

### CeRNA Network Construction

The ceRNA networks, for each CRC site, were constructed using the GDCRNATools v1.6 package of R, and the DE lncRNAs, miRNAs, and mRNAs. The network is based on the mRNA-miRNA-lncRNA interactions predicted by the spongeScan (20) algorithm and the starBase v2.0 (21) database. In the ceRNA networks, positively correlated mRNAs and lncRNAs act as sponges by sharing a significant number of miRNA binding sites and suppressing their functioning. The ceRNA networks generated show the possible molecule interactions related to each CRC site.

### Functional Analysis

The functional analysis to assess the biological processes in the ceRNAs was done with the enrichment module of GDCRNATools. The gene list used for Gene Ontology (GO) (22), Kyoto Encyclopedia of Genes and Genomes (KEGG) (23), and Disease Ontology (DO) (24) came from the org.Hs.eg.db database v3.11.4, and all the human pathways from KEGG were considered. The adjusted p-value ≤ 0.05 was set as the threshold for statistical significance for GO, KEGG, and DO. Although some pathways present FDR > 0.05, they were still included as they represent good discussion points for the CRC functional analysis.

### Survival Analysis

The Cox Proportional-Hazards (CoxPH) model from GDCRNATools was used to calculate the hazard ratio (HR) of the ceRNA molecules. An outlier removal was performed and only molecules with |higherLimit − HR| ≤ 6 and |lowerLimit − HR| ≤ 6 were considered. The survival curve was constructed by using the Kaplan Meier (KM) analysis from GDCRNATools. CoxPH and KM were used p < 0.05 as the threshold for statistical significance.

## Results

### Differentially Expressed RNAs

**Figure 1:**
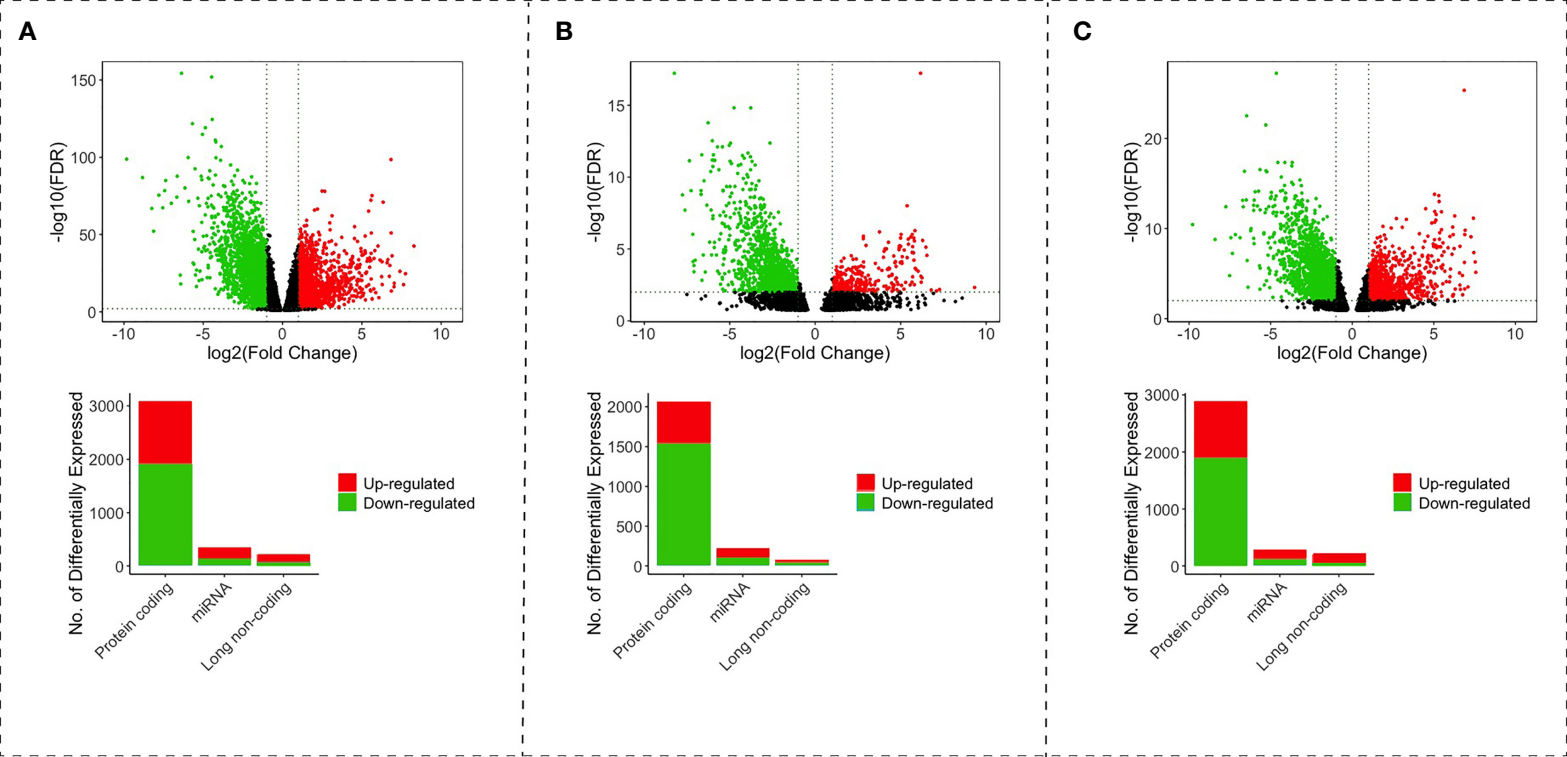
Volcano and bar plot with differentially expressed lncRNAs, miRNAs and mRNAs of colon **(A)**, rectum **(B)**, and rectosigmoid junction **(C)** sites. The red points and bars represent upregulated RNAs. The green points and bars represent downregulated RNAs.

We obtained a total RNA expression raw count of 541 cancer and 48 non-tumor samples from 539 patients with CRC from the TCGA-COAD and TCGA-READ projects. A differential expression analysis was performed for each type of CRC. In the colon analysis, we found 140 upregulated and 75 downregulated lncRNAs, 213 upregulated and 136 downregulated miRNAs, and 1,179 upregulated and 1,906 downregulated *PCGs* (**Figure 1A**). In the rectum, we found 46 upregulated and 37 downregulated lncRNAs, 119 upregulated and 99 downregulated miRNAs, and 535 upregulated and 1,532 downregulated *PCGs* (**Figure 1B**). In the rectosigmoid junction, we found 149 upregulated and 59 downregulated lncRNAs, 181 upregulated and 108 downregulated miRNAs, and 1,005 upregulated and 1,880 downregulated *PCGs* (**Figure 1C**).

### CeRNA Networks

**Figure 2:**
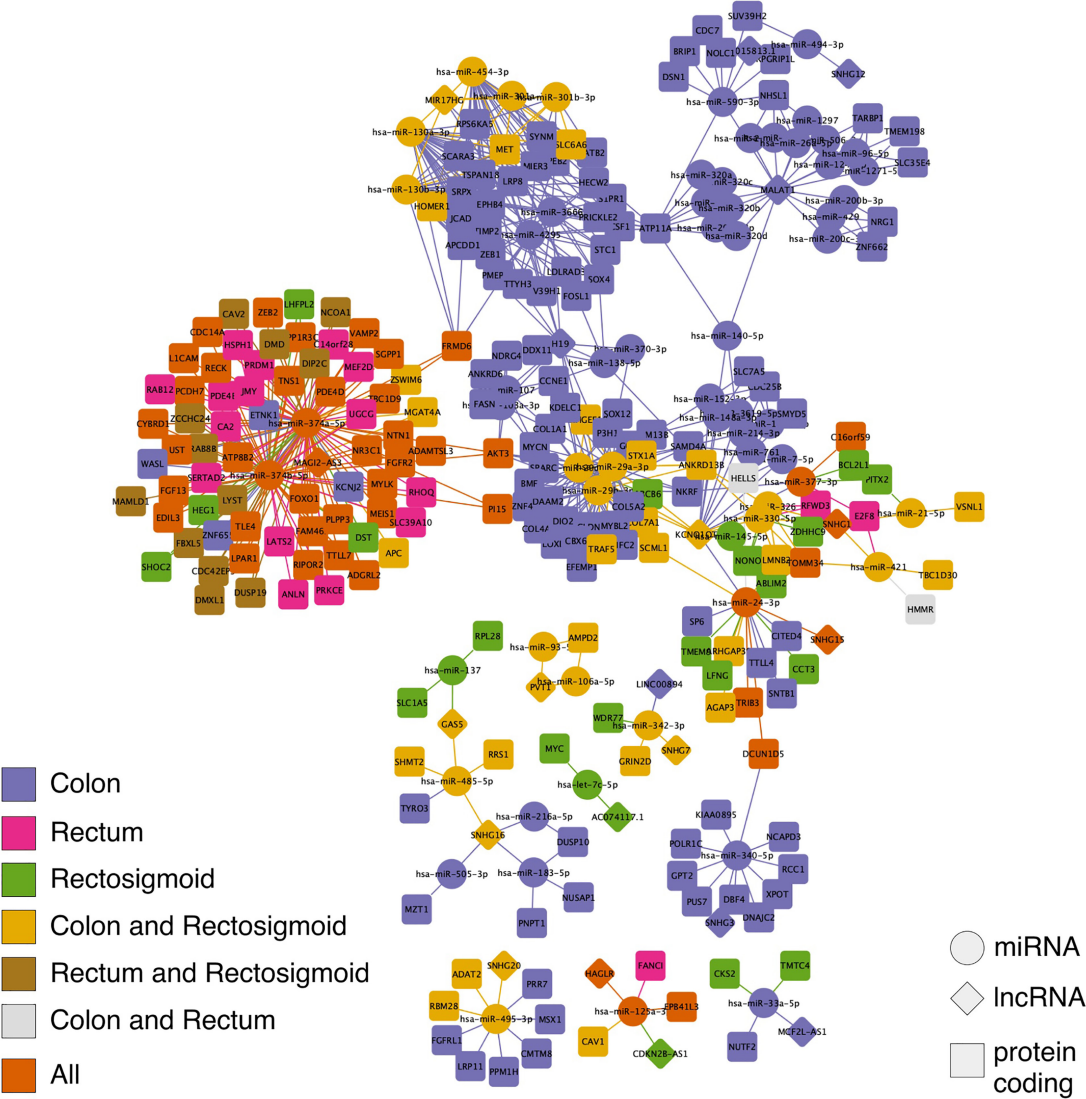
Competing endogenous RNA (ceRNA) network in colon, rectum and rectosigmoid junction sites. The diamonds represent lncRNAs, the circles represent miRNAs, and the squares represent *PCGs*. The molecules and interactions of each CRC site can be identified by color.

A ceRNA network, represented by a graph with its vertex (nodes) representing the molecules and the lines connecting them representing the interactions between the molecules, was established for each of the CRC sites. For colon, a ceRNA network consisting of 239 nodes and 506 interactions was established. For rectum, a ceRNA network consisting of 79 nodes and 136 interactions was established. For rectosigmoid junction, a ceRNA network consisting of 131 nodes and 210 interactions was established. We also analyzed the intersection between the ceRNA networks and found that: colon and rectum share 2 nodes and 2 interactions; colon and rectosigmoid junction share 48 nodes and 77 interactions; rectum and rectosigmoid junction share 12 nodes and 23 interactions; and all three sites share 47 nodes and 76 interactions. Furthermore, we performed an analysis to find individual nodes and interactions for each CRC site, finding that: colon has 142 nodes and 351 unique interactions; rectum has 18 nodes and 35 unique interactions; and rectosigmoid junction has 24 nodes and 34 unique interactions. **Figure 2** shows all the described networks.

**Figure 3:**
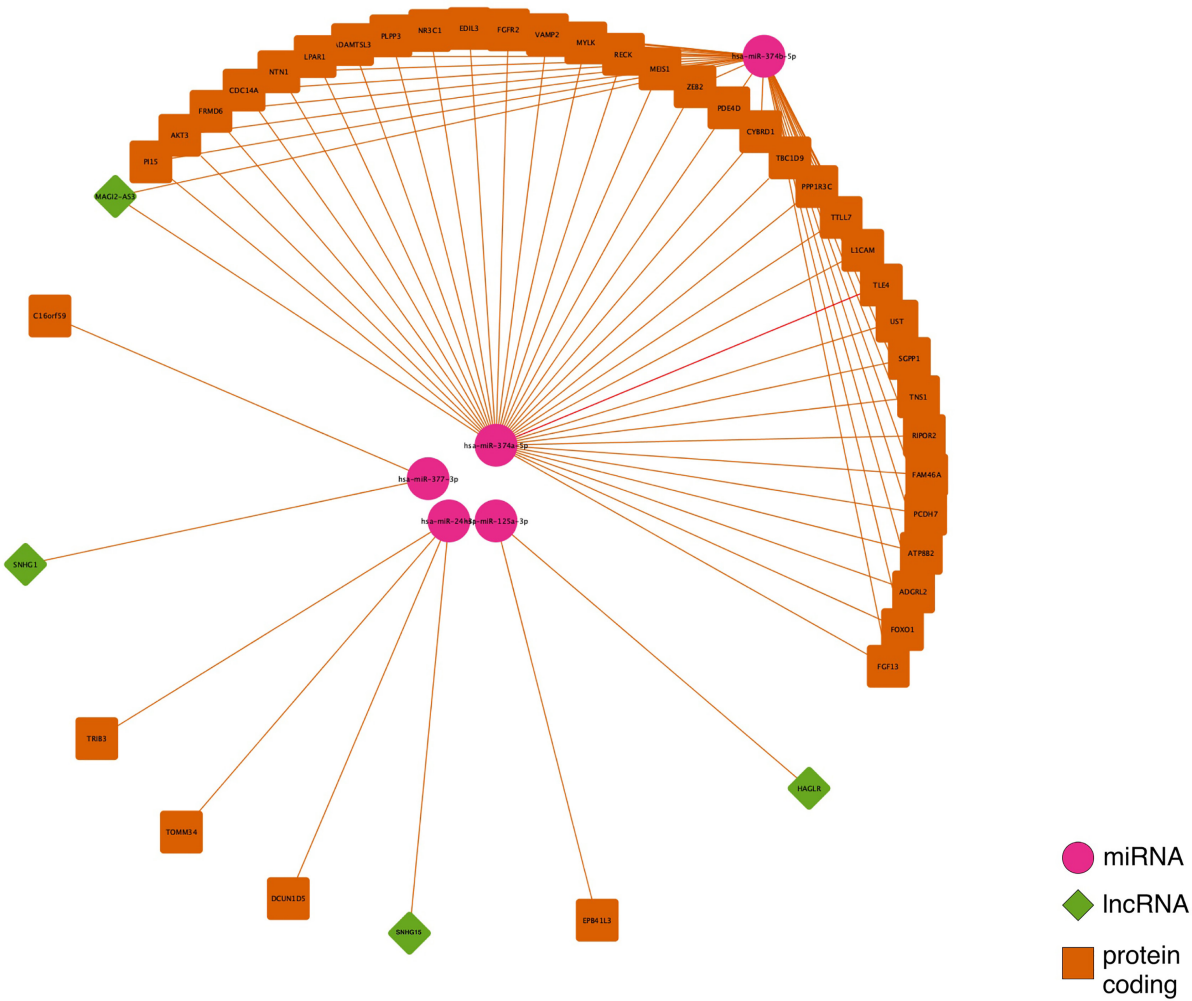
Competing endogenous RNA (ceRNA) network intersection for colon, rectum and rectosigmoid junction sites.

The intersection between each of the three site-specific ceRNA networks is regulated by the lncRNAs: *MAGI2-AS3*, *HAGLR-AS3*, *SNHG1*, and *SNHG1*5 (**Figure 3**). *HAGLR-AS3*, *SNHG1*, and *SNHG1*5 compose three different small ceRNA networks by sponging hsa-miR-125a-3p, hsa-miR-377-3p, and hsa-miR-24-3p, respectively. In the *MAGI2-AS3* network, we see the ceRNA mechanism affecting a great number of *PCGs via* hsa-miR-374b-5p and hsa-miR-374a-5p, suggesting that this lncRNA has a great impact on the main CRC mechanism, which occurs at all sites.

### Functional and Survival Analysis

**Figure 4:**
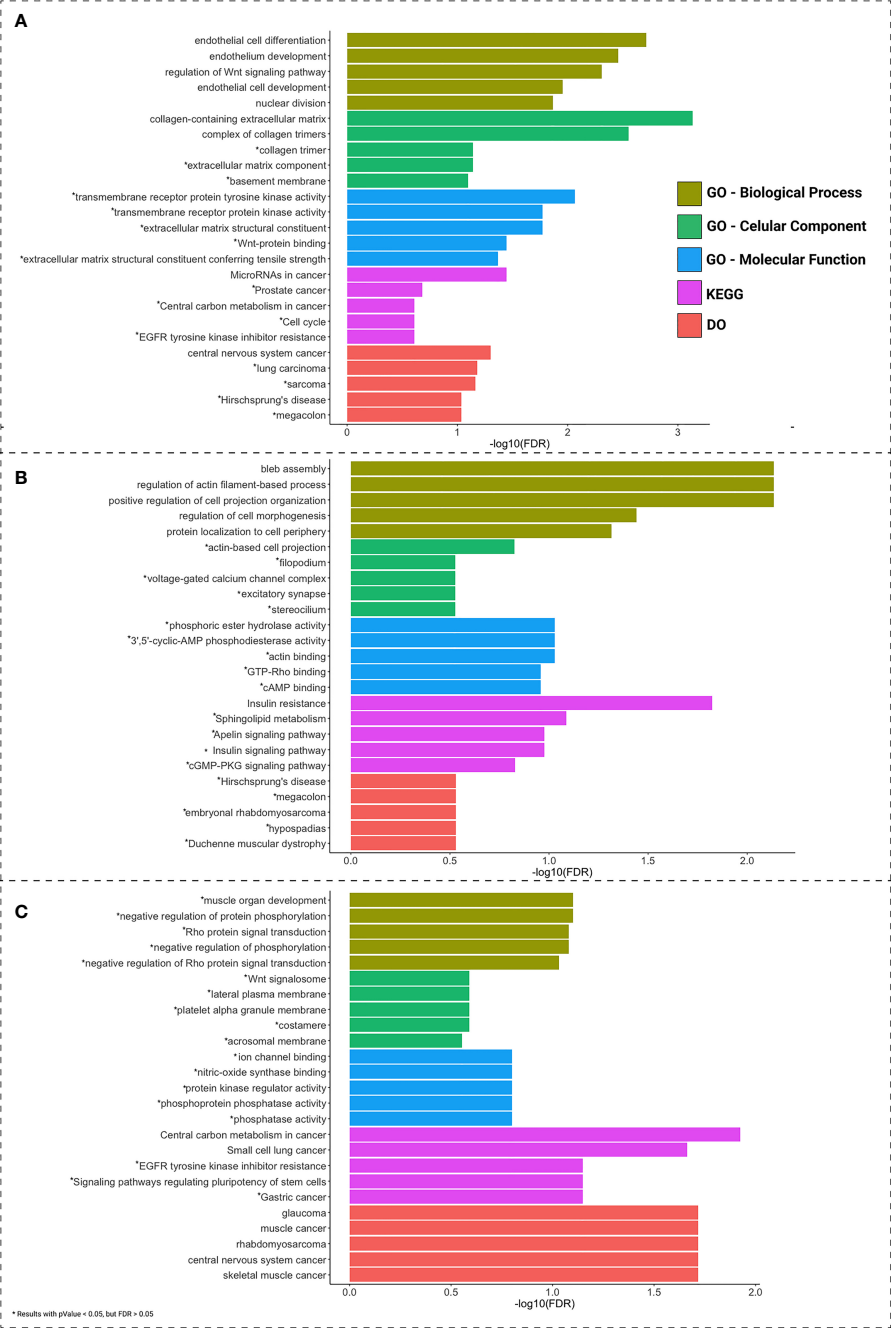
Functional enrichment analysis of *PCGs* and lncRNAs included in the ceRNA network of colon **(A)**, rectum **(B)**, and rectosigmoid junction **(C)** sites. The top 5 enrichment results for GO biological processes, cellular component, molecular function, DO and KEGG are shown in different colors. Asterisks (*) indicate pathways presenting FDR > 0.05.

A functional enrichment analysis was performed with GO, KEGG, and DO, to indicate the potential biological roles of the ceRNAs, lncRNAs, and *PCGs* (**Figure 4**). Each of the sites presented a different main functional characteristic: colon pathways are mainly related to endothelial differentiation; rectum pathways are mainly related to apoptosis; and rectosigmoid junction pathways are mainly related to signal transduction.

**Figure 5:**
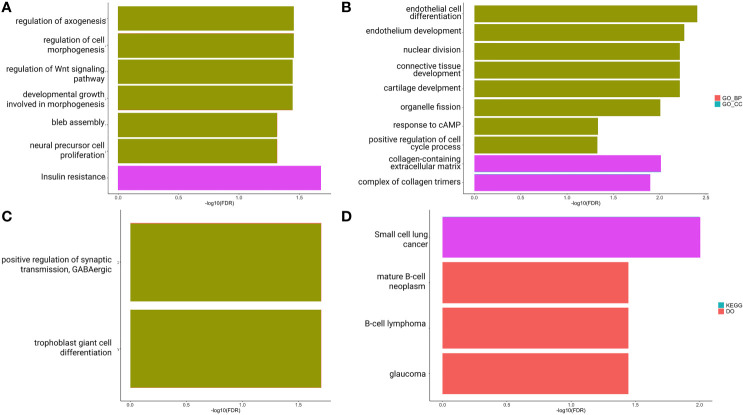
Functional enrichment analysis of *PCGs* and lncRNAs included in the ceRNA network: common to colon, rectum and rectosigmoid junction **(A)**; specific to colon **(B)**; specific to rectum and **(C)**; specific to rectosigmoid junction **(D)** sites.

To better understand the impact of ceRNA networks, we also performed a functional enrichment analysis for the *MAGI2*-*HAGLR-AS3*-*SNHG1*-*SNHG15* intersection ceRNA network (**Figure 3**), and for each site-specific ceRNA network. The molecules present in the ceRNA network common in all CRC sites are mainly related to cell morphogenesis pathways (**Figure 5A**), such as regulation of Wnt signaling and cell morphogenesis and the process of insulin resistance. In the colon-specific (**Figure 5B**) ceRNA network, the most significant pathways are involved in tissue differentiation and development, such as endothelial cell differentiation and connective tissue development, while the rectum-specific (**Figure 5C**) ceRNA network is related to cell differentiation. No pathways were significant for the rectosigmoid junction site (**Figure 5D**).

**Figure 6:**
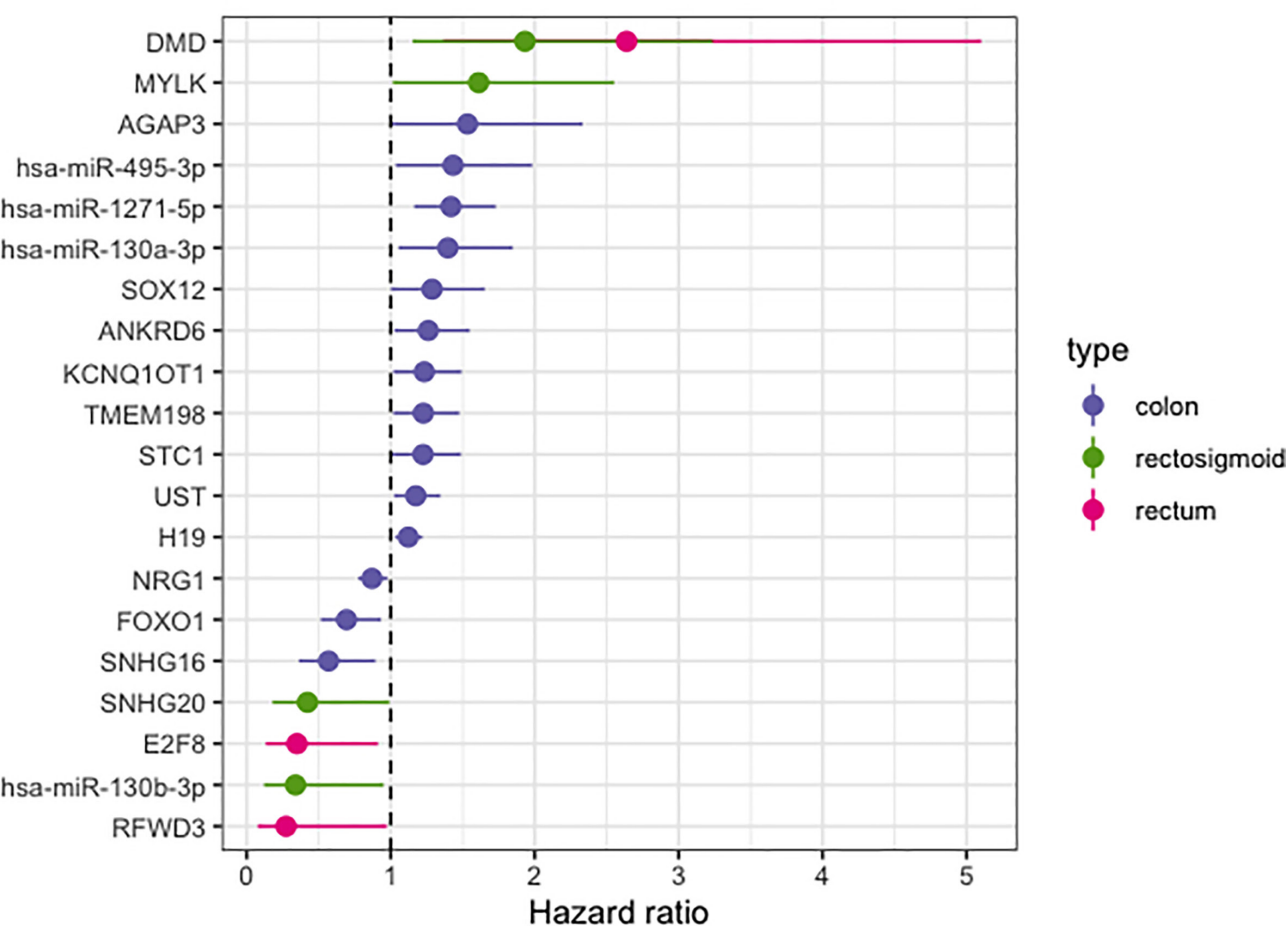
Hazard ratio forest plot of survival associated RNAs in the ceRNA network for colon, rectum and rectosigmoid junction sites. The molecules with hazard ratio < 1 indicate risk factors, and those with hazard ratio > 1 indicate protective factors.

Clinical data from CRC patients was used to obtain the HR and to build the overall survival time curve. For each CRC site, we had data from 391 colon, 84 rectum, and 66 rectosigmoid junction patients. Using CoxPH, we identified 20 molecules from the previous ceRNA networks with relevant HR, being 14 in colon, 3 in rectum, and 4 in rectosigmoid junction molecules (**Figure 6**). The *DMD* gene was the only molecule with high HR, present in more than one CRC site.

**Figure 7:**
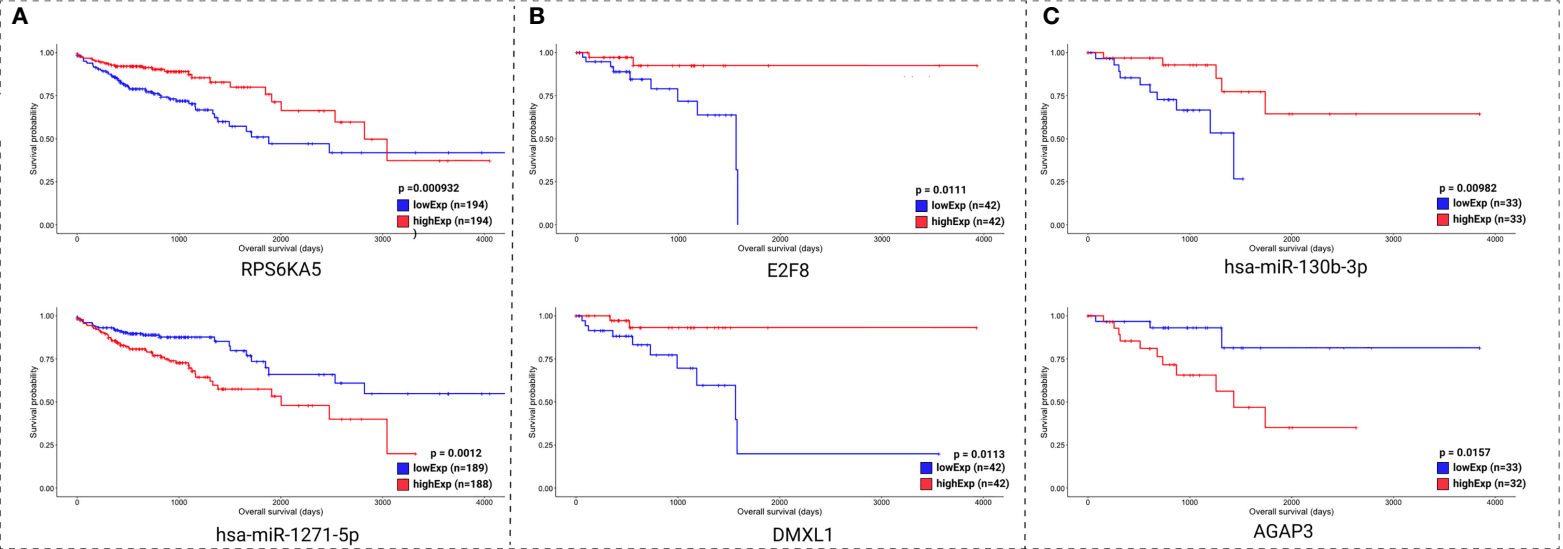
Kaplan-Meier survival curves for the two best scored molecules for colon **(A)**, rectum **(B)**, and rectosigmoid junction **(C)** sites. Horizontal axis: overall survival time (in days). Vertical axis: survival probability.

**Figure 8:**
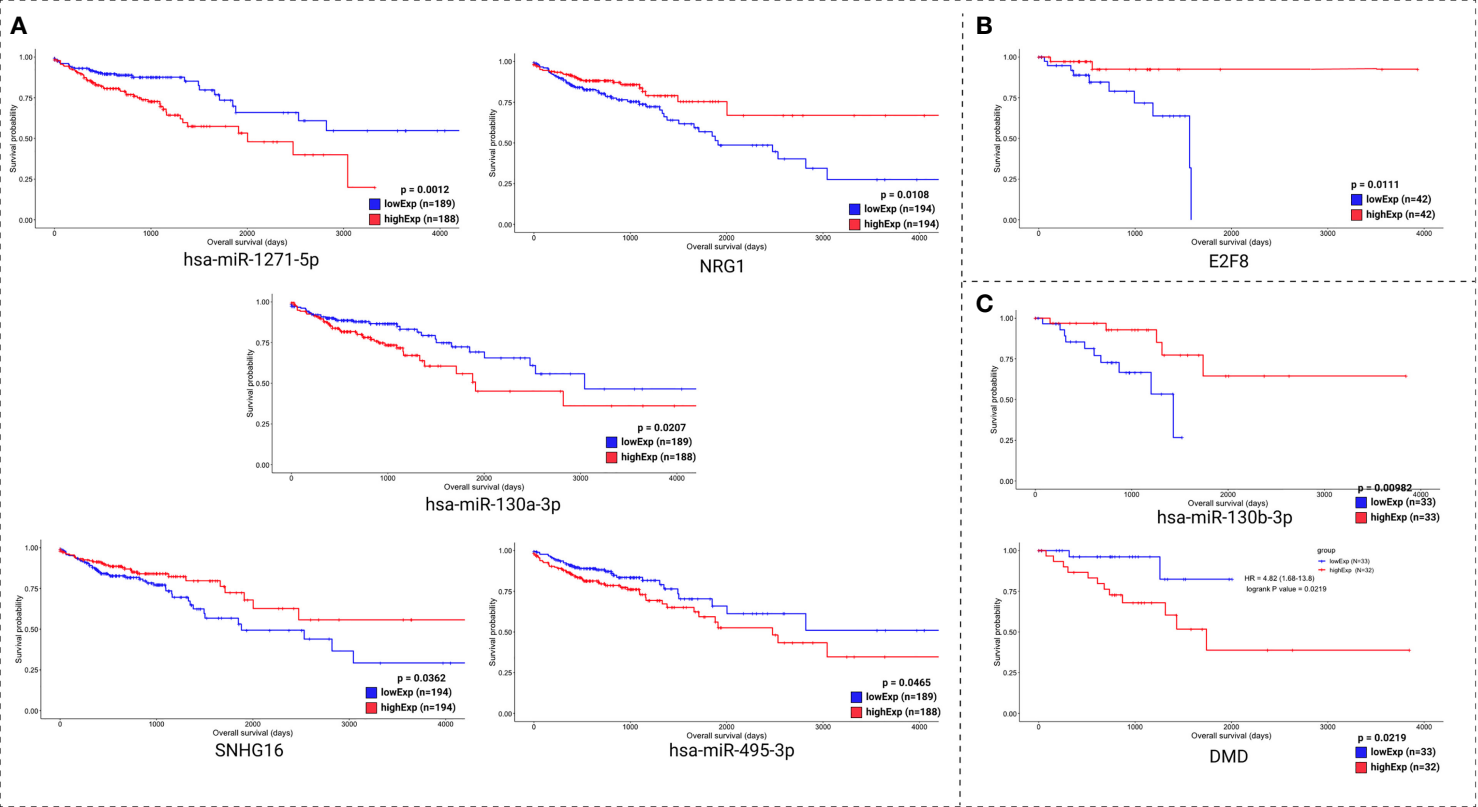
Kaplan-Meier survival curves for the best scored molecules with top HR from CoxPH for colon **(A)**, rectum **(B)**, and rectosigmoid junction **(C)** sites.

Clinical data and the expression profile of the HR relevant genes were used for overall survival analysis. These analyses were divided into two sets: the molecules with lowest pValue according to the KM method (**Figure 7**) and the molecules with lowest pValue according to the KM and COxPh methods (**Figure 8**).

Regarding the KM method (**Figure 7**), we found some *PCGs* that were not in the group of 20 molecules found with CoxPH (**Figure 6**), but are relevant for overall patient survival, such as *RPS6KA5* for colon, *DMXL1* for rectum, and *AGAP3* for rectosigmoid junction.

Regarding the CoxPH method (**Figure 7**), we identified molecules that can be considered potential biomarkers for patient prognosis. The CoxPH methodology also revealed several molecules to be significant for overall survival in each anatomical site (**Figure 8**), many of which are concurrent with KM method results, such as has-miR-1271-5p, *E2F8*, and hsa-miR-130-3p.

**Figure 9:**
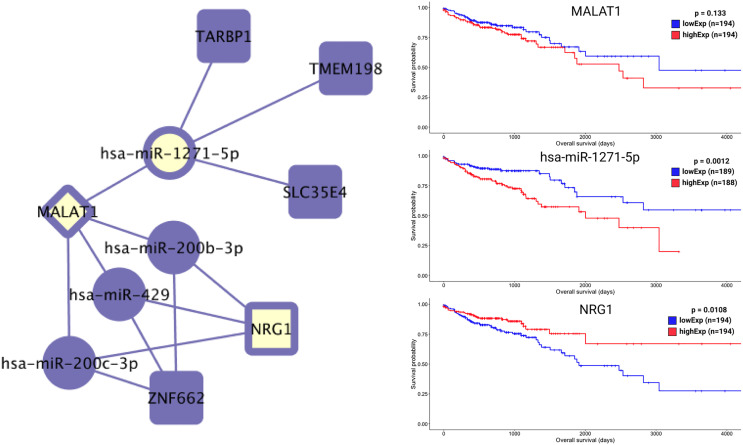
Colon-specific ceRNA network and overall survival for hsa-mir-1271-5p, *NRG1* and MALAT1. Here, we can see the opposing putative roles of hsa-mir-1271-5p and *NRG1* on overall survival time. MALAT1 is shown as a link that relates hsa-mir-1271-5p and *NRG1* regulation, as well as behaving like hsa-mir-1271-5p on patient overall survival time.

Most of the relevant molecules for colon cancer patient survival (**Figures 7** and **8**) are present in distinct regions of the ceRNA network, with the exception of miR-1275-5p and *NRG1*. These two molecules are connected by MALAT1 ceRNA (**Figure 9**).

## Discussion

CRC is one of the most common and lethal cancers worldwide. The treatment therapy is directly related to tumor location. Due to heterogeneous characteristics, tumors located in the rectosigmoid junction are treated as either colon or rectum tumors. Therefore, obtaining molecular markers that could help identify tumor site and molecular characteristics is necessary. In this sense, ceRNA networks allow us to evaluate differentially expressed molecules as well as miRNA-lncRNA-PCG interactions and control mechanisms.

In this study, we used differentially expressed lncRNAs, *PCGs*, and miRNAs to build specific ceRNA networks for the anatomical sites most affected by CRC and identify different and common factors involved in the progression of CRC. Moreover, we further accessed the relevance of each of these molecules in the ceRNA in regard to their impact on prognosis and their functional implications.

Previous works (11, 15, 16) have reported a protagonist role for the lncRNA *H19* as protagonist in regulating various cancer-related mRNAs in colon cancer and in CRC in general. However, in our analysis, *H19* is only present in the colon exclusive network and not in that of the rectum, also described by Zhiyuan et al. (14). In this network, *H19* presents itself as a risk factor and acts as ceRNA for *SOX12*, *ANKRD6*, *STC1*, and hsa-miR-130a-3p, all of which are present as putative risk factors.

Colon, rectum and rectosigmoid junction presented common ceRNA networks regulated by the lncRNAs *MAGI2-AS3*, *HAGLR-AS3*, *SNHG1*, and *SNHG1*5, suggesting some similarities in CRC development independent of the anatomical site (**Figure 3**). These common mechanisms are related to the regulation of Wnt signaling, cell morphogenesis, and proliferation (**Figure 5A**), which are known to be present in cancer. The lncRNAs regulating common ceRNA networks were already correlated with known cancer pathways, such as: MAGI2-AS3 with cell apoptosis and proliferation in CRC (25); *HAGLR-AS3* with cell proliferation, invasion, and apoptosis (26); *SNHG1* with cell growth and promotion of CRC through the Wnt/β-catenin signaling pathway (27, 28); and SNHG15 with cell proliferation, apoptosis, and activation of the Wnt/β-catenin signal in CRC (29, 30). These studies indicate *MAGI2-AS3*, *HAGLR-AS3*, *SNHG1*, and *SNHG15* as potential biomarkers involved in regulation of Wnt signaling, cell morphogenesis, and proliferation. Our study is the first to bring together all of these molecules and related ceRNA networks as common factors in all CRC sites and to indicate their joint use as potential biomarkers for colon, rectum, and rectosigmoid junction cancer common behavior. Within the *MAGI2-AS3* network, we found the dystrophin gene (*DMD*). *DMD* plays a special role in muscle fiber integrity (31), and it was the only gene identified as a potentially significant risk factor in both rectum and rectosigmoid junction sites. Duchenne muscular dystrophy is a disease known to be associated with *DMD*, and our functional analysis relates the biological disease’s pathways from DO to the rectum ceRNA (**Figure 4B**). This gene is part of a network where it is regulated by miRNAs hsa-miR-374a-5p and hsa-miR-374b-5p, and the lncRNA *MAGI2-AS3*. These three ncRNAs connected to DMD are also ‘sponged’ by the PCG FOXO1, which is critical to tumor suppression and apoptosis (32) and presented a putative protective role in colon CRC tumors. Although Zhong et al. (11) previously reported their interaction, the authors did not mention the *DMD* and FOXO1 genes, nor did they evaluate their putative role as biomarkers or as survival factors. Therefore, to the best of our knowledge, this is the first time that *DMD* is reported as a potential biomarker for poor prognosis in CRC. In the case of the rectosigmoid junction, we found *DMD* and hsa-miR-130b-3p to be relevant to patient prognosis. Some studies have reported the importance of hsa-miR-130b-3p in poor prognosis of CRC (33, 34). It is worth noting that hsa-miR-130b-3p, which is relevant to the rectosigmoid junction is in the same ceRNA network as hsa-miR-130a, which is relevant to colon prognosis. Both molecules are regulated by the lncRNA MIR17HG, which may indicate that this ceRNA network is relevant to both colon and rectosigmoid junction. However, the miRNA responsible for poor patient prognosis is different for each site.

The specific networks for colon and rectum present distinct enriched biological pathways, with more specific endothelial development in the colon and cell morphology in the rectum. Due to the low number of samples for the rectosigmoid junction, we were unable to find a statistically significant pathway for this network. However, the pathways found are related to phosphorylation and signal transduction. These different biological pathways highlight differences in CRC behavior between distinct anatomical sites, thus reinforcing the importance of correctly identifying the tumor site.

*E2F8* and *RFWD3* presented putative protective roles for rectum CRC tumors. *E2F8* encodes transcription factors that regulate development by the cell cycle (35), and *RFWD3* is known to be essential in the process of repairing DNA interstrand cross-links (36). Both genes are connected with the lncRNA *SNHG1* but are regulated by different miRNA. The *SNHG1* ceRNA network is common for all CRC sites, but only interacts with *RFWD3* and *E2F8* in the rectum, indicating a potential role for this network in rectum cancer. The *E2F8* gene has been reported as relevant to CRC as well as in regulating cancer progression (35, 37) and our survival analysis indicates better survival for high *E2F8* expression levels. Previous studies (35, 37) have identified *E2F8* as a biomarker for colon cancer, but they did not evaluate the potential role of *SNHG1*-*RFWD3*-*E2F8* ceRNA network interaction in rectum cancer.

The *RPS6KA5* gene encodes for a tyrosine kinase and has been indicated as a biomarker for colon cancer (38) through interaction with hsa-miR-130a (39). In our colon-specific network, the lncRNA MIR17HG sponges hsa-miR-130a and interacts with *RPS6KA5*. Hsa-miR-1271-5p, hsa-miR-130a, *SOX12*, *ANKRD6*, *TMEM198*, *STC1*, *H19*, and *NRG1* all presented potential risk factors for colon cancer. Most of these molecules are present in distinct regions of the ceRNA network, with the exception of miR-1275-5p and *NRG1*. Both of these molecules are connected to the lncRNA MALAT1 and present opposing putative roles (**Figure 9**). Some studies (11, 15, 16) have previously reported the effects of *H19* ceRNA on CRC, but both our network and survival analyses suggest its influence only in the case of tumors located in the colon. No enrichment pathway of the rectosigmoid junction presented an exclusive HR relevant molecule.

In further consideration of the overall survival evidence, we reaffirm the potential role as prognosis biomarkers for hsa-miR-1271-5p, *NRG1*, hsa-miR-130a-3p, *SNHG16*, and hsa-miR-495-3p, in the colon; *E2F8*, in the rectum; and of *DMD* and hsa-miR-130b-3p, in the rectosigmoid junction.

This study had some limitations. Firstly, although several novel lncRNAs, *PCGs*, and miRNAs with clinical significance for CRC were found, the study was performed with TCGA data and no further experimental validation was carried out. Secondly, less information was analyzed for rectum and rectosigmoid junction tissue than for that of colon, which could influence site-specific results. Research on ceRNAs in CRC is still in development and requires further experimental studies and greater amount of data from colon, rectum, and rectosigmoid cancer in order to improve our understanding of the biomarkers found.

In conclusion, this study constructed a ceRNA network for colon, rectum, and rectosigmoid that provides clinical significance and functional implications for cancer at each of these sites. The results indicate several potential prognostic markers for colon, rectum, and rectosigmoid cancer, and also suggest that the ceRNAs found can help explain the differences between, and common factors on, prognosis for these CRC sites.

## Data Availability Statement

The raw data supporting the conclusions of this article will be made available by the authors, without undue reservation.

## Author Contributions

LV contributed to conception and design of the study, wrote the manuscript, and performed the analysis. NJ collaborated and reviewed the study on the bioinformatics methods and biology assumptions. JCS, MW, and PS reviewed and collaborated to key points at the discussion and methodology on a bioinformatics perspective. JBS reviewed and collaborated to key points at the discussion and methodology on a medical perspective. All authors contributed to the article and approved the submitted version.

## Conflict of Interest

The authors declare that the research was conducted in the absence of any commercial or financial relationships that could be construed as a potential conflict of interest.
